# Do Individual Differences and Aging Effects in the Estimation of Geographical Slant Reflect Cognitive or Perceptual Effects?

**DOI:** 10.1177/2041669516658665

**Published:** 2016-07-18

**Authors:** Abigail M. Dean, Jaehyun Oh, Christopher J. Thomson, Catherine J. Norris, Frank H. Durgin

**Affiliations:** Department of Psychology, Swarthmore College, PA, USA

**Keywords:** 3D perception, slant perception, spatial cognition, individual differences, aging

## Abstract

Several individual differences including age have been suggested to affect the perception of slant. A cross-sectional study of outdoor hill estimation (*N* = 106) was analyzed using individual difference measures of age, experiential knowledge, fitness, personality traits, and sex. Of particular note, it was found that for participants who reported any experiential knowledge about slant, estimates decreased (i.e., became more accurate) as conscientiousness increased, suggesting that more conscientious individuals were more deliberate about taking their experiential knowledge (rather than perception) into account. Effects of fitness were limited to those without experiential knowledge, suggesting that they, too, may be cognitive rather than perceptual. The observed effects of age, which tended to produce lower, more accurate estimates of hill slant, provide more evidence that older adults do not see hills as steeper. The main effect of age was to lower slant estimates; such effects may be due to implicit experiential knowledge acquired over a lifetime. The results indicate the impact of cognitive, rather than perceptual factors on individual differences in slant estimation.

The perception of the slants of hills has been the focus of a great deal of research in the past 20 years. Very large distortions are evident in the conscious perception of slant (Kammann, 1967; Li & Durgin, 2009; Proffitt, Bhalla, Gossweiler, & Midgett, 1995; Ross, 1974; Shaffer & Flint, 2011). A hill of 5° is typically perceived as being 20°; conversely, a hill that looks to be 30° might actually be only 8°. Models of slant perception taking viewing distance into account have revealed that perceived slant is exaggerated at all viewing distances (even near small surfaces appear more slanted from horizontal than they should; Durgin, Li, & Hajnal, 2010), but that perceived slant increases with the log of viewing distance (Bridgeman & Hoover, 2008; Li & Durgin, 2010).

There has been controversy over whether there exists a separate accurate representation of slant that can be measured by motor actions (Creem & Proffitt, 1998, 2001; Durgin, Hajnal, Li, Tonge, & Stigliani, 2010, 2011). A preponderance of evidence now suggests that haptic perception of slant is just as exaggerated as visual perception (Durgin, Hajnal et al., 2010; Durgin & Li, 2012; Hajnal, Abdul-Malak, & Durgin, 2011). Systematic measurement biases such as anchoring (Feresin, Agostini, & Negrin-Salviolo, 1998; Shaffer, McManama, & Durgin, 2015; Shaffer, McManama, Swank, Williams & Durgin, 2014), postural effects (Li & Durgin, 2011), and reference frame bias (Coleman & Durgin, 2014) may all contribute to the cognitive illusion that manual measures (e.g., palm boards) tap into an accurate representation of hill slant (Durgin, Hajnal et al., 2010). In general, it appears possible that because action is calibrated to perception, stable distortions in perception can effectively support the control of action without requiring accuracy (e.g., Durgin, 2009, 2014). The exaggeration of perceived slant relative to horizontal may therefore be an adaptive coding strategy that is useful even in the control of action (Durgin, 2009; Durgin & Li, in press).

Some of the most puzzling results concerning slant perception are the individual differences that have been reported based on sex (Proffitt et al., 1995) and age (Bhalla & Proffitt, 1999). In many studies, it has been found that slant estimates of hills are higher for female participants than for male participants, but it has not been clear whether this reflects a (perceptual) gender difference in how hills actually appear, or if it reflects a (cognitive) gender difference in the cognitive transformation of perceptual experience into a spatial or numeric response. Second, the claim has been advanced that hills appear steeper to older adults (Bhalla & Proffitt, 1999). However, the data originally reported in support of this claim appear to better support the idea that older adults give lower (i.e., more accurate) estimates of hill slant (perhaps because of greater life experience; see Durgin, Hajnal et al., 2010, for a review): Bhalla and Proffitt’s older participants only gave relatively high estimates for a couple of very steep hills for which no direct comparison data were available from younger participants, whereas for hills of 4° to 10° (which include fairly challenging, but walkable slopes), the data of Bhalla and Proffitt (1999; Table 2) consistently show that older adults gave lower, more accurate, estimates than did younger participants. Recent data for small, near surfaces have also suggested that slant estimates given by older participants are also lower and more accurate than the estimates given by younger participants (Norman, Crabtree, Bartholomew, & Ferrell, 2009).

One of the concerns that has complicated the interpretation of individual differences in slant perception is that the results of estimation tasks can be biased by subtle or not-so-subtle instructional manipulations (see Durgin et al., 2009; Durgin, Hajnal et al., 2010). For example, the data from older adults reported by Bhalla and Proffitt (1999) were collected in a very different experimental design than the “normative” data to which it was compared. Moreover, they were preceded by instructions that might be said to invoke stereotype threat (Steele & Aronson, 1995) or experimental demand (Durgin et al., 2009). That is, older participants were first asked how hard the hill would be to climb for someone of their age and physical ability. This initial question might well have affected older adults’ subsequent estimates (e.g., Woods, Philbeck, & Danoff, 2009) for very steep hills. The younger participants in the normative study (Proffitt et al., 1995) to which Bhalla and Proffitt’s data from older adults were compared had not been asked any such (potentially biasing) question before making their estimates, so any comparison of the two sets of data ought to be done with caution.

Similarly, studies involving fitness, fatigue, or burden manipulations (Bhalla & Proffitt, 1999) may also tap into individual differences in combination with biases introduced by experimental demand. Durgin et al. (2009) and Durgin, Klein, Spiegel, Strawser, and Williams (2012) found that most participants asked to wear a heavy backpack during a perception experiment assumed the backpack was intended to affect the slant estimates they were asked to make while wearing the backpack. Participants asked to do strenuous exercise in advance of making slant estimates (e.g., Bhalla & Proffitt, 1999; Proffitt et al., 1995) probably draw similar inferences about the experimenter’s intent. Durgin et al. (2009) found that only a subset of participants indicated that they had cooperated with the experimental demand, and this subset showed reliably higher slant estimates (see also Shaffer, McManama, Swank & Durgin, 2013). Bhalla and Proffitt reported that individual differences in physical fitness affected estimates of hill slant. However, their study of this confounded physical fitness with sex, which rendered the results hard to interpret (see Durgin et al., 2010, for discussion).

To try to minimize experimental demands of direct manipulation of fitness or age (Durgin et al., 2009; Firestone, 2013; Firestone & Scholl, in press), we conducted an exploratory cross-sectional study on slant perception. We sought to be able to model our data not only with respect to individual differences, such as age and gender, but also with respect to other forms of individual difference, such as personality variables that covary with sex (Schmitt, Realo, Voracek, & Allik, 2008), as well as cognitive differences, such as knowledge of slant bias (Durgin & Li, 2011; Stigliani, Li, & Durgin, 2013). A personality variable like *agreeableness*, for example, is sex-linked, such that females tend to be higher than males on this trait (Schmitt et al., 2008). If many participants guess that experimenters want them to give high estimates, but more agreeable participants are more likely to cooperate with this implicit demand, a sex difference in slant estimation (but not slant perception) could result from this personality trait. By testing for effects of agreeableness in a variable population, it may be possible to test whether sex differences in slant estimation might arise from trait differences. Moreover, by sampling an off-campus population in a multidimensional study along with on-campus participants, we sought to minimize experimenter bias effects associated with null hypothesis testing.

To maximize sensitivity to population variables (individual differences), our study focused primarily on a single hill, which was assessed with manual, visual, and verbal measures. All participants were first tested with this hill. In a classic study reporting sex differences, Proffitt et al. (1995) collected three measures similar to these at a single hill for each participant. We devoted our entire *N* to a single hill, though two further hills were tested with verbal measures. Our primary analysis is of the perceived slant of a paved path on a single steep (9°) hill.

## Methods

### Participants

One-hundred and six adults (46 males), naïve to the purpose of the study, were successfully recruited to participate in our study from the student body at Swarthmore College or from the surrounding community. Forty-eight of the participants were nonstudent community members, ranging in age from 18 to 72 years old (15 males); 58 of the participants were Swarthmore College students, ranging in age from 18 to 22 (31 males). Recruitment materials indicated that participants were to wear corrective lenses if they needed them, that participants must be able to speak and read English, and that they must be able to stand while making their judgments. Visual acuity was measured using a Snellen chart for all but one participant (with whatever corrective lenses they wore during the experiment); only two participants had (corrected) vision worse than 20/40 (one was 20/50; one 20/70). The study methods were approved by the local institutional review board.

### Hills

**Figure 1.**
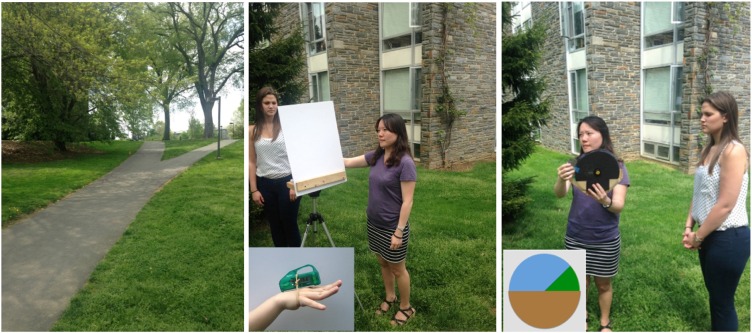
The primary, 9°, hill (left) and the two nonverbal measures depicted in use.

Three hills were tested in the same order for each participant. The primary hill, shown in Figure 1, left panel, is a steep campus path with a slant of 9° as measured by an inclinometer at the location marked by the orange cone off to one side. The second hill was a very steep grassy embankment at the side of a nearby path, with a slant of 22.5°. The third hill was a broad campus path with a slant of 4.5°. The to-be-judged portion of each hill was marked with either a small orange cone (hills 1 and 2) or by an existing landmark to the side of the path (hill 3). Participants stood at a fixed location when making their estimates from the base of each hill, and the portion of the path or embankment to be judged was roughly at eye level.

### Measures of Perceived Slant

Three measures were used for the primary (9°) hill. The measures were collected in a fixed order designed to minimize the risk of measure-to-measure bias. Participants were first asked to indicate the slant of the hill by holding their hand parallel to the hill (they held their hand up behind a vertical screen so that they could not see their hand). They wore a lightweight inclinometer on their hand so that we could measure the orientation of the back of their hand. We also measured the angular thickness of their hand when they placed it on a horizontal level surface after holding it up. The calculated orientation of the midline axis of their hand relative to horizontal was used as measure of their intended orientation (see Li & Durgin, 2011). We call this the manual matching response (MMR).

The second measure used was based on the visual measure developed by Proffitt et al. (1995). In this case, the participant had to adjust a 2D depiction of a hill (see Figure 1, right panel). An estimate in degrees (relative to horizontal) was then read off a digital protractor mounted on the measure. This will be called the visual matching response (VMR). It was always started from a slant of 0°.

Finally, a verbal estimate was requested in degrees relative to horizontal. This measure was collected last so that it would be less likely to influence the nonverbal measures. The reasoning for this was that if a participant first gave a verbal response, they might be likely to try to match their visual and manual responses to the verbal response, which would be easier to remember than would be the manual and visual matches. We call this the numeric estimation response (NER). Note that for the 22.5° and 4.5° hills only an NER was collected.

### Measures of Individual Differences

To avoid influencing slant judgments, all demographic information and other measures of individual differences were collected after the slant judgments were completed. An assessment of visual acuity was first performed using a Snellen eye chart viewed from 6.1 m. After this, all of the individual difference information was collected on computer by a custom program implemented in E-Prime.

The personality variables were tested first. These were three scales of the Big Five Inventory that are known to be sex-linked (Schmitt et al., 2008): Female participants tend to score higher than male participants on agreeableness (AG) and conscientiousness (CO) and lower on emotional stability (ES). Each scale consisted of 20 items. Participants indicated the degree to which a trait word described them on a 9-point scale ranging from *not at all* (1) to *extremely* (9). Sample items for agreeableness, conscientiousness, and emotional stability are (a) “helpful,” (b) “practical,” and (c) “moody” (reverse coded), respectively. Note that a positivity bias might be expected for such scales, but such a bias would predict high correlations across all three scales, and this was not observed.

We intended to measure spatial ability by including a computerized mental rotation task with Shepard and Metzler (1971) shapes (Peters & Battista, 2008). There is a great deal of evidence for sex differences in spatial tasks (e.g., D. Voyer, S. Voyer, & Bryden, 1995) including those related to slope (Nardi, Holmes, Newcombe, & Weisberg, 2015; Nardi, Meloni, Orlandi, & Olivetti-Belardinelli, 2014). However, data from this reaction time task, which involved judging whether each of two imaged shapes was the same or different from another, were reliably correlated both with age and with conscientiousness.^1^ This rendered its value as a measure of spatial ability questionable; it was not used for analysis.

Participants were next asked whether they had prior experiences or special knowledge that was relevant to the evaluation of hill slant (we gave as examples experience with skiing, measuring a slant with an inclinometer, or having had a driveway repaved); 36 participants listed some form of such experience. We also asked participants whether they used any particular strategies in making their judgment, though this data were intended simply to screen for unusual approaches to the task and were not included in statistical analyses.

Finally, we collected demographic information such as sex and age, and also self-reported weight and height, which was used to estimate body mass index (BMI_SR_). BMI has been used previously as a marker of fitness by proponents of embodied perception (e.g., Bhalla & Proffitt, 1999; Sugovic & Witt, 2011). We used de-identified self-report of weight to avoid privacy concerns where some of our participants were peers of the experimenters. Because we wondered if high-heeled footwear might influence slant judgments, we also asked them to indicate the height of the heels of their shoes, though there turned out to be insufficient variance in this measure to use it as a predictor.

The correlations among our various predictor variables are shown in Table 1. It can be seen that the personality traits we expected to be sex-linked did not strongly covary with sex in our sample, but that they did all tend to covary with age, with a particularly strong correlation (*r* = .38) between age and emotional stability, (*p* < .0001).

**Table 1.** Correlation Coefficients Between Individual Difference Measures.

	Knowledge	Age	Sex	AG	CO	ES
Age	0.061					
Sex	–0.042	0.077				
AG	0.249	0.205	0.086			
CO	0.081	0.204	–0.054	0.060		
ES	0.026	0.379*	–0.072	0.221	0.238	
BMI_SR_	–0.123	0.223	–0.037	0.055	0.022	0.097

*Note.* AG = agreeableness; CO = conscientiousness; ES = emotional stability; BMI = body mass index.

* *p* < .0001.

**Table 2.** Model for Those With Knowledge About Slant (*N* = 39).

	Estimate	Standard error	*t* value	*p* value
Intercept	20.44	1.08	18.937	<.0001***
Age	0.08	0.07	1.194	.2420
Sex	3.03	2.03	1.490	.1466
AG	1.94	1.52	1.280	.2105
CO	–3.53	0.88	4.003	.0004 ***
ES	–1.48	0.89	1.673	.1047
BMI_SR_	–0.13	0.27	0.501	.6203

*Note.* AG = agreeableness; CO = conscientiousness; ES = emotional stability; BMI = body mass index. *** < .001

### Procedure

Once participants arrived at the lab, they signed a consent form and were paid in advance for their time. All participants were first involved in a separate study of distance perception and height perception in a field near the Psychology building (Oh et al., 2015). Following this, participants were driven in a golf cart by an experimenter to a different part of campus where they stopped at each of the three hills, made their five judgments (three at the first hill and one at each of the other two) and were then driven back to the Psychology building where the individual difference measures were collected.

### Analysis

As has been reported previously (Shaffer, McManama, Swank, et al., 2014; Stigliani et al., 2013), all three of the slant measures were reliably correlated with each other, suggesting a common underlying representation: MMR and VMR, *r*_104_ = 0.50, *p* < .001; MMR and NER, *r*_104_ = 0.29, *p* = .003; VMR and NER, *r*_104_ = 0.54, *p* < .001. The three types of measure were therefore averaged to produce a single estimate of perceived hill slant for the 9° hill for each participant (note that separate analyses are also reported for each measure below for completeness). We then used simultaneous multiple linear regression modeling to look at all of the data from the first hill using the individual difference scores as predictors.

## Results

In previous studies (e.g., Durgin & Li, 2011), prior experiential knowledge relevant to slant estimation (e.g., experience with skiing) has been a predictor of slant estimates, and the present data replicate this observation. A simultaneous multiple linear regression model was tested that included knowledge, conscientiousness, agreeableness, emotional stability, age, sex, and BMI_SR_ as predictors, as well as all two-way interactions between them (all variables were first centered). This analysis showed main effects of knowledge, sex, and BMI_SR_: Those reporting some knowledge gave lower (more accurate) estimates, *β* = −5.12°, *t*(74) = 2.52, *p* = .014, *R*^2 ^= 0.05; female participants gave higher slant estimates, *β* = 4.15°, *t*(74) = 2.32, *p* = .023, *R*^2 ^= 0.04; and participants with higher BMI_SR_ gave higher slant estimates, *β* = 0.50°, *t*(74) = 2.35, *p* = .022, *R*^2 ^= 0.06. However, the analysis also showed that knowledge interacted both reliably with conscientiousness, *t*(74) = 2.77, *p* = .007, and marginally with BMI_SR_, *t*(74) = 1.83, *p* = .072. We therefore split the data according to whether the participants reported having knowledge relevant to slant (*N* = 39) or not (*N* = 67).

### Reliable Predictors in the Presence of Knowledge: Conscientiousness

**Figure 2.**
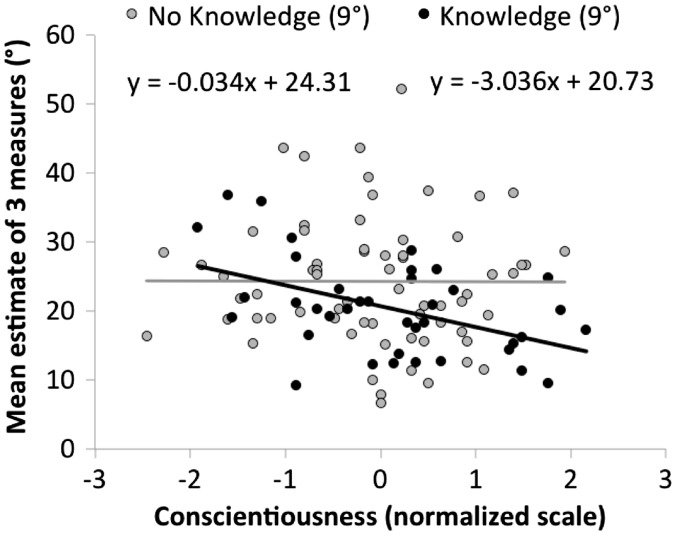
Interaction between conscientiousness and knowledge on estimates of 9° hill.

A simultaneous multiple linear regression model of the responses of those reporting any experiential knowledge relevant to slant (with age, sex, all three personality factors, and BMI_SR_ as predictors) is shown in Table 2. The only reliable predictor of estimates among knowledgeable people was conscientiousness, which predicted reliably lower (and thus more accurate) slant estimates, *β* = −3.53, *t*(30) = 4.00, *p* < .001, *R*^2 ^= 0.23. Although this result was not anticipated, this pattern is consistent with the idea that knowledgeable people scoring higher in conscientiousness were more likely to take the trouble to use their knowledge (rather than merely their perceptual experience) when asked to provide a slant estimate. A plot depicting the interaction between conscientiousness and knowledge is shown in Figure 2.

### Reliable Predictors in the Absence of Knowledge

For those who reported no particular knowledge about slant (*N* = 67), a simultaneous multiple linear regression model, shown in Table 3, indicated three reliable predictors: age, sex, and BMI_SR_. We will consider each of these observations in turn.

**Table 3.** Model for Those With No Knowledge of Slant (*N* = 67).

	Estimate	Standard error	*t* value	*p* value
Intercept	23.41	1.03	22.686	<.0001***
Age	–0.20	0.07	2.928	.0049**
Sex	4.23	2.09	2.028	.0471*
AG	–0.35	1.22	0.290	.7729
CO	0.17	1.02	0.163	.8713
ES	0.46	1.04	0.440	.6617
BMI_SR_	0.69	0.20	3.372	.0013**

*Note.* AG = agreeableness; CO = conscientiousness; ES = emotional stability; BMI = body mass index. * < .05, ** < .01, *** < .001

#### Age

**Figure 3.**
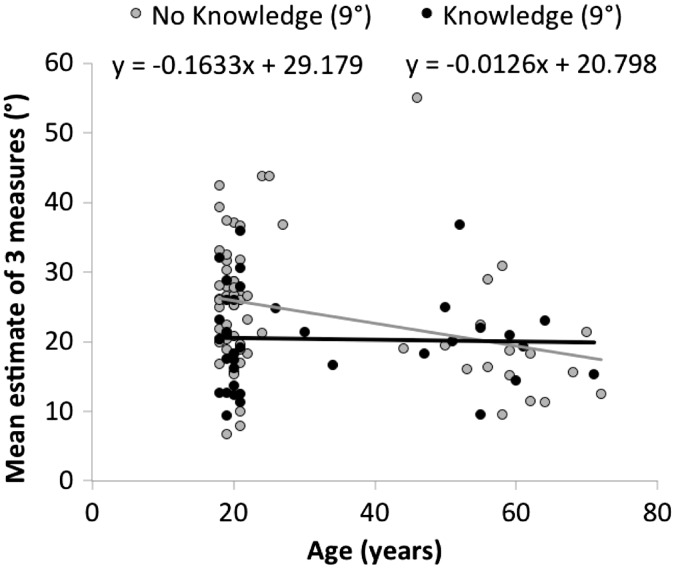
Age effect on estimates of 9° hill among is present among those without knowledge, but not among those reporting experiential knowledge.

As shown in Figure 3, older participants gave estimates that were lower than those of younger participants by about 2° per decade, *β* = −0.20°/year, *t*(59) = 2.93, *p* = .005, *R*^2 ^= 0.084. The direction of this effect differs from the claims of Bhalla and Proffitt (1999) who argued that older people saw hills as steeper. However, for a hill of a similar magnitude to this one (as well as for all lower hills they tested), Bhalla and Proffitt found a similar pattern: Older adults in their study gave lower estimates than younger observers (see also Norman et al., 2009). Thus, we have replicated the finding that older adults are more accurate than are younger adults in slant estimation of a typical walkable slope. It remains possible that these effects reflect an accumulation of world knowledge that is not detected by our experiential knowledge probe.

We note that measured visual acuity was, of course, correlated with age, but the age effect found here remained even when visual acuity was included in the model as a covariate.

#### Sex

Consistent with previous reports, female participants gave estimates that were 4.2° higher than male participants, *t*(59) = 2.03, *p* = .047, *R*^2 ^= 0.034. The present data suggest that these effects are not due to sex-linked personality differences (which were included in the model), nor by the forms of experiential knowledge measured by our probe. Note that excluding sex as a factor in this model did not unmask any effects of sex-linked personality traits.

#### BMI_SR_

**Figure 4.**
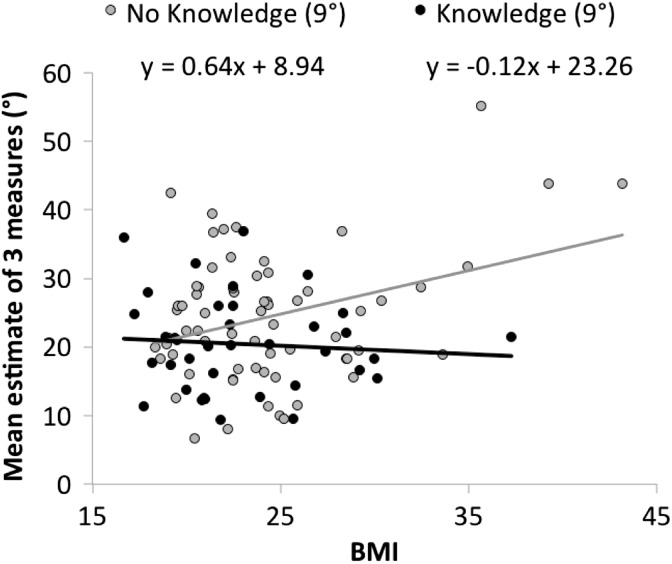
BMI_SR_ effect on estimates of 9° hill among those without knowledge is driven by obese participants (i.e., BMI > 30). BMI = body mass index.

As shown in Figure 4, participants with higher BMI_SR_ gave higher slant estimates, *β* = 0.69, *t*(59) = 3.37, *p* = .001, *R*^2 ^= 0.132. This appears to lend support to the claim that behavioral potential (i.e., fitness) affects slant perception. However, a closer look at the data here shows that the effect is limited to obese *women*, which may support the alternative perspective that knowingly having a particularly high BMI may influence how participants make their estimate.

Of the 67 people in the no-knowledge condition, seven can be classified as obese, and it is a subset of these seven that drive the BMI_SR_ effect: If we eliminate the seven obese participants (without knowledge) from our analysis, not only does BMI_SR_ cease to be predictive, *t*(52) = 0.13, but sex is no longer predictive either, *t*(52) = 1.10.

Thus it seems possible that greater salience of body image among females is responsible for at least a portion of the sex differences found in previous studies. Among the nine obese participants in our overall sample, the six females gave reliably higher average estimates of the 9° hill (*M* = 38.3°) than did the three males (*M* = 18.6°), *Welch t*(6.3) = 4.08, *p* = .006. If the influence of obesity on slant judgment is sex-linked, this would seem consistent with the idea that effects of obesity are based on sociocognitive factors rather than perceptual effects.

#### Differences between measures?

There is a controversial claim in the literature that the three measures we used tap into different underlying representations (e.g., Creem & Proffitt, 1998, 2001; but see Durgin, Hajnal et al., 2010). In particular, Creem and Proffitt suggested that manual measures tap into dorsal (“accurate”) processing streams, whereas visual and verbal measures reflect conscious perception. Shaffer et al. (Shaffer, McManama, & Durgin, 2015; Shaffer, McManama, Swank, et al., 2014) have argued that manual measures are just more susceptible to anchoring effects.

To address this issue here, we tested whether manual measures were immune to individual differences. As we expected, however, given that the three measures are typically correlated, regression models computed for each of the three measures individually showed evidence of effects of age and of BMI_SR_ among those without knowledge of slant.

The pattern among those with knowledge of slant differed somewhat for the manual measure in a manner that might also indicate a role of sex-linked personality traits. The first estimate given by each participant was the manual estimate. When the manual estimates were analyzed separately, they showed evidence of an interaction between knowledge and agreeableness (rather than between knowledge and conscientiousness). In fact, among those reporting some knowledge of slant, manual estimates tended to increase with agreeableness, *β* = 3.98°, *t*(30) = 2.02, *p* = .052, *R*^2 ^= 0.115. Although only a marginal result, statistically, this is exactly the sort of pattern (an interaction of knowledge and agreeableness) that one would predict of participants who might be trying to co-operate with what they expect the experiment to be about (Durgin et al., 2009). Insofar as their knowledge gave them some awareness that hill slant tends to be overestimated, those participants who were more agreeable tended to give higher estimates in their very first estimate, perhaps believing that the experimenters wished to measure overestimation of hill slant.

This evidence that agreeable participants were tempted to “help” initially, is not inconsistent with the further observation, that, when finally asked to give a verbal estimate, the conscientious participants (among those who had knowledge) now gave reliably lower verbal estimates, *β* = −7.06°, *t*(30) = 4.46, *p* < .001, *R*^2 ^= 0.258. That is we see evidence that both agreeableness and conscientiousness interact with the presence of some knowledge about hill slant overestimation in determining responses.

Because the order in which the different types of estimates were collected was fixed, we cannot draw strong conclusions about whether this is indeed an order effect or an effect of the manual nature of the measure. However, the present data are consistent with the idea that personality variables can interact with background knowledge of participants both to elevate estimates (including manual estimates) and to rein them in.

#### Replication with the steep embankment

For the 22.5° embankment, a preliminary analysis of verbal estimates showed a similar pattern to the data from the primary hill. There was a marginal effect of knowledge, *β* = −2.81°, *t*(74) = 1.83, *p* = .071, and a reliable interaction between knowledge and conscientiousness, *t*(74) = 2.02, *p* = .048. A separate analysis of the participants reporting some knowledge about hill slant indicated a marginal effect of conscientiousness, *β* = −4.76°/unit, *t*(30) = 1.93, *p* = .064, consistent with the results for the primary hill. The analysis of those without knowledge (Table 4) replicated the effects of age, *β* = −0.25°/year, *t*(59) = 2.16, *p* = .035, found for the primary hill, as well as (marginally) the higher estimates of females than males, *β* = 7.26°, *t*(59) = 1.99, *p* = .051. However, the effect of BMI_SR_ was not reliable for this hill, *β* = 0.34°, *t*(59) = 0.98, *p* = .332. Given that the same participants gave judgments at the primary hill first, the replication of most of the patterns is reassuring, but perhaps unsurprising since participants were likely trying to remain consistent with their previous estimate.

**Table 4.** Model for those with no knowledge of slant (N = 67) for the steep (22.5°) embankment.

	Estimate	Standard error	*t* value	*p* value
Intercept	45.78	1.80	25.393	<.0001***
Age	–0.25	0.12	–2.163	.0346*
Sex	7.26	3.65	1.991	.0512
AG	1.84	2.13	0.862	.3923
CO	1.08	1.78	0.609	.5451
ES	–1.66	1.82	0.914	.3646
BMI_SR_	0.35	0.36	0.977	.3324

*Note.* AG = agreeableness; CO = conscientiousness; ES = emotional stability; BMI = body mass index * < .05, ** < .01, *** < .001.

#### Replication with the shallow path

For the 4.5° path, analysis of verbal estimates again showed a reliable interaction between knowledge and conscientiousness, *t*(74) = 2.34, *p* = .022. However, a separate analysis of the participants reporting some knowledge about hill slant did not detect any reliable main effects. The analysis of those who reported no knowledge showed a marginal effect of BMI_SR_, *β* = 0.34°/unit, *t*(59) = 1.86, *p* = .068, while no other main effects were reliable. It seems likely that individual difference effects from the previous analyses were not detectable at this hill simply because the mean estimates were compressed relative to the variance.

One way of expressing differential proportional variance is the coefficient of variation (CoV), which is the standard deviation of a measure divided by the mean. For the verbal estimates of the 4.5° hill, the CoV was 0.69, which was substantially greater than the 0.36 CoV for verbal estimates of the 22.5° hill, *F*(105, 105) = 3.69, *p* < .001, and also much higher than the 0.38 CoV of the combined estimates for the 9° hill, *F*(105, 105) = 3.37, *p* < .001.

Note that CoVs of the individual measures for the 9° hill were somewhat higher than those of the combined response: They ranged from 0.43 for the visual matches to 0.52 for the verbal estimates, whereas combining these correlated measures effectively produced a more sensitive measure by reducing proportional variance. Durgin, Hajnal et al. (2010) have discussed differential proportional variance as an artifactual source of apparent dissociation between different types of slant measure. The current result illustrates how differential proportional variance can also affect analysis of data from different hills. The results also indicate the advantage of combining correlated measures into a single measure for the purpose of reducing error variance.

#### How does aging affect slant perception? A comparison to the findings of Bhalla and Proffitt (1999)

**Figure 5.**
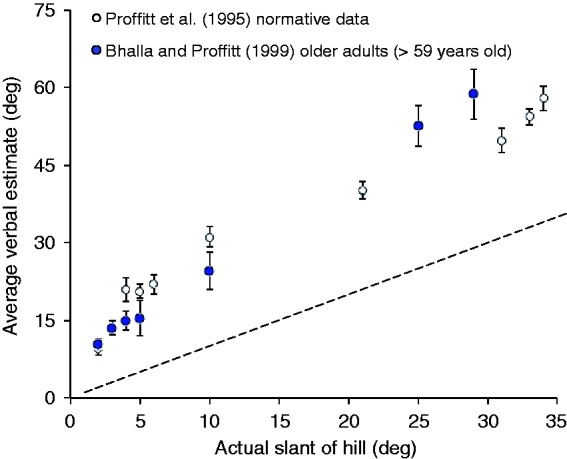
Verbal estimation data published by Bhalla and Proffitt (1999; Table 2) arguing that older adults see hills as steeper than do younger adults. Note that their conclusion appears to be contradicted in the range from 4° to 10°. Standard errors of the means are shown.

Bhalla and Proffitt (1999) collected hill slant estimation data from older participants (age 60 and above). Each older participant in their study saw four hills. They compared these data with normative data collected in a prior study (Proffitt et al., 1995) with different sets of hills, a different design, and different instructions. They concluded from this comparison that older adults saw hills as steeper. However, their own data seem to contradict this conclusion for the majority of hill slants tested, and their design leaves the comparisons between the two groups risky overall. The verbal estimation data used by Bhalla and Proffitt are replotted in Figure 5. It can be seen that for hill slants of 4°, 5°, and 10°, their older participants gave lower estimates than were provided in the normative data. Proffitt and Bhalla adopted the rather unusual statistical approach of comparing estimates that were averaged across the entire range of 2° to 10° and reported a null statistical effect.

**Figure 6.**
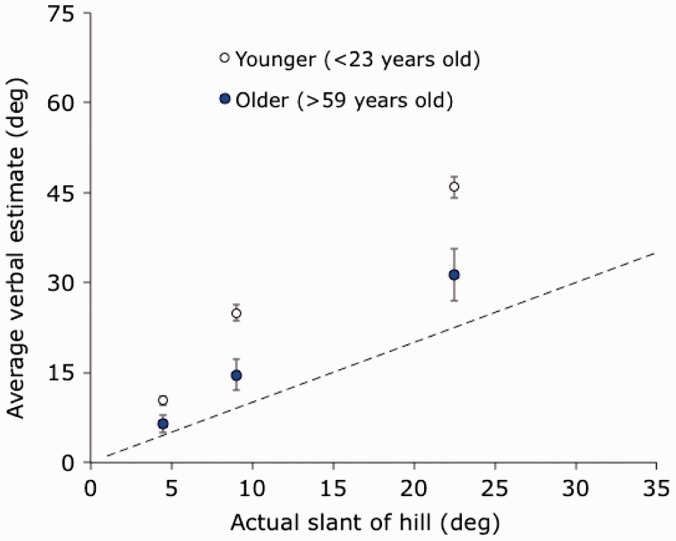
Verbal estimation data for the three hills we tested, divided into older and younger adults. Standard errors of the means are shown.

For comparison with their results, we computed average verbal estimates at each of the three hills we tested for all participants older than 59 years old (*N* = 11) as well as for participants younger than 23 years old (*N* = 70). These data are shown in Figure 6 along with standard error bars. At each of the three hills we tested, the estimates of the older participants (*M* = 6.5°, 14.6°, 31.4°) are less than the estimates of the younger participants (*M* = 10.3°, 25.0°, 46.0°), *Welch t*(16.1) = 2.30, *p* = .035, *Welch t*(16.0) = 3.57, *p* = .003, *Welch t*(13.7) = 3.08, *p* = .008, respectively.

In summary, older adults gave lower (more accurate) estimates than younger adults for all the hills we tested including a very steep embankment. Note that there is evidence that older adults are also more accurate at estimating egocentric distance (Bian & Anderson, 2013) and exocentric depth extents (Norman, Adkins, Norman, Cox, & Rogers, 2015), and knowledge effects have also been implicated for estimates of distance (Durgin, Leonard-Solis, Masters, Schmelz, & Li, 2012). The present age effect on slant estimation is consistent with cognitive factors playing a significant role in slant estimation. We did not see any evidence that older adults gave higher slant estimates, as had been proposed by Bhalla and Proffitt (1999). Even for our steepest hill (22.5°), the older adults gave lower estimates than did the younger adults. It might be objected that our sample was self-selected and thus cannot support strong conclusions about effects of aging. For example, Bhalla and Proffitt recruited some of their older participants from a senior living community, and their participants may have differed from ours in health and fitness. These concerns, however, ought to be mitigated by the observation that our data seem to agree with the majority of the data of Bhalla and Proffitt (as well as the data of Norman et al., 2009 collected for small surfaces) in suggesting that older adults are generally more accurate at estimating slant than are younger adults. The two hills where the data of Bhalla and Proffitt suggest otherwise were not tested with younger participants and were tested with potentially biasing instructions.

## Discussion

Our study sought to evaluate individual differences in slant estimation in order to differentiate between cognitive (e.g., modularist; Firestone & Scholl, 2016) and perceptual (e.g., embodied perception; Bhalla & Proffitt, 1999) interpretations of such individual differences. Two of the strongest and most consistent predictors we observed were age and experiential knowledge. Experiential knowledge could involve a variety of activities, such as skiing, that might allow one to learn about the mismatch between perceived and actual slant. More than a third of our population reported some experiential knowledge, and these participants gave lower, more accurate, estimates of hills. Age acted similarly in that older participants gave lower, more accurate judgments that were similar to those who explicitly reported having experiential knowledge (see Figure 3).

Of particular importance, we found that conscientiousness could have a substantial role in estimating slant: Among participants explicitly indicating some experiential knowledge concerning slant, conscientiousness seemed to predict whether they would use that knowledge to lower (improve) their estimates both for the steep path and for the steep embankment. This observation helps to show that cognitive effects of experience play an important role.

We found only circumstantial evidence that sex differences in slant estimation might be due to personality differences in our study. First, for those with some knowledge, agreeableness seemed to elevate initial manual slant estimates. This is consistent with the idea that participants who suspected that we wanted to get overestimates tried to oblige us to the extent that they were agreeable. Although agreeableness was not strongly correlated with sex in our study, it is generally regarded as a sex-linked trait, such that females tend to score higher on measures of agreeableness than males. A combined role of agreeableness and knowledge might be a pathway whereby females would end up giving higher estimates than males even though their perceptual experience might not differ on average.

A second hypothesis for explaining sex-differences in hill slant estimation was suggested by our findings related to obesity. Obesity was a predictor of slant estimates among those who did not report having any special knowledge of slant. However, this effect was limited to female participants. Future investigations may want to consider whether obese female participants are more likely to provide particularly high estimates for social reasons or because they actually perceive hills as geometrically steeper. That is, it seems unlikely that these participants really meant that the 9° hill appeared to be tilted half way to vertical; perhaps their exaggerated estimates reflect stereotype threat or consciously taking imagined effort into consideration (Woods et al., 2009).

Overall, our data suggest that age, knowledge, and personality differences produced cognitive rather than perceptual effects in this study. The effect of age, in particular, went contrary to the widely cited claims of embodied perception. We have suggested that those claims probably ought not to have been made in the first place given that the original data for older participants were collected under such different circumstances than the data of younger participants to which it was compared. The effect of experiential knowledge, by seeming to remove age and fitness effects, strongly suggests that the age effects we have documented are more cognitive than perceptual. That is, experiential knowledge did not simply act as an additional factor, nor did it produce particularly accurate estimates on average, but it seemed to replace these other factors rather than combine with them.

Although there may additionally be individual differences in the perceived slant of hills, it is hard to measure such individual differences because cognitive factors can easily overwhelm perceptual variation. The subject-wise correlations across measures we replicated here might mean that participants are reporting their individual and unique percept by each of the measures. However, correlations could also emerge as the result of individual differences in the cognitive translation processes from perception to a reportable impression.

More striking to us than these possible individual differences is the success of recent quantitative models of hill perception, such as the angular scale expansion model proposed for hill perception (Li & Durgin, 2010, 2012). This model uses one fixed parameter (an angular gain of 1.5, estimated across a variety of studies; Durgin & Li, 2011) and one free parameter that accounts for an additional logarithmic effect (see also Bridgeman & Hoover, 2008) of viewing distance on slant perception measures both for explicit slant estimation (e.g., the classic hill data of Proffitt et al., 1995) and implicit (shape-based) measures of perceived slant (Li & Durgin, 2010, 2012). These models propose that the striking overall bias observed in slant perception may represent a species-wide functional (efficient) perceptual coding strategy that effectively exaggerates the (shallow) range of slants most often encountered in perceptual experience.

The present findings regarding effects of age, knowledge, and personality suggest that a complete model of cognitive processes of slant estimation might profitably include a variety of postperceptual transformations and adjustments. These postperceptual processes clearly involve cognitive effects of experience and knowledge that appear to interact with personality variables such as conscientiousness and agreeableness. Depending on context, estimation may additionally be susceptible to social and cognitive biases including anchoring, stereotype threat, and experimental demand characteristics—which may also interact with personality variables.

## Note

1 Simultaneous multiple regression of mental rotation latency and accuracy on other predictors showed that each was predicted by both age (latency: *t*(97) = 5.37, *p* < .001; accuracy: *t*(97) = 3.16, *p* = .002) and by conscientiousness (latency: *t*(97) = 2.02, *p* = .046; accuracy: *t*(97) = 2.25, *p* = .027).
